# The short-lived African turquoise killifish: an emerging experimental model for ageing

**DOI:** 10.1242/dmm.023226

**Published:** 2016-02-01

**Authors:** Yumi Kim, Hong Gil Nam, Dario Riccardo Valenzano

**Affiliations:** 1 Max Planck Institute for Biology of Ageing, D50931, Cologne, Germany; 2 Department of New Biology, DGIST, 711-873, Daegu, Republic of Korea; 3 Center for Plant Aging Research, Institute for Basic Science, 711-873, Daegu, Republic of Korea

**Keywords:** Ageing, Longevity, Age-associated diseases, Model organisms, Turquoise killifish, *Nothobranchius furzeri*

## Abstract

Human ageing is a fundamental biological process that leads to functional decay, increased risk for various diseases and, ultimately, death. Some of the basic biological mechanisms underlying human ageing are shared with other organisms; thus, animal models have been invaluable in providing key mechanistic and molecular insights into the common bases of biological ageing. In this Review, we briefly summarise the major applications of the most commonly used model organisms adopted in ageing research and highlight their relevance in understanding human ageing. We compare the strengths and limitations of different model organisms and discuss in detail an emerging ageing model, the short-lived African turquoise killifish. We review the recent progress made in using the turquoise killifish to study the biology of ageing and discuss potential future applications of this promising animal model.

## Abstract (precis)

**Summary:** The short-lived African turquoise killifish is an emerging experimental vertebrate model organism, ideal for studies aimed at discovering the basic mechanisms underlying vertebrate ageing and adult phenotypes.

## Introduction

Biological ageing consists of a wide range of dynamic changes that occur throughout an organism's lifespan that negatively impact all fundamental biological processes and eventually result in the loss of organismal homeostasis and, ultimately, lead to death (Kirkwood and Austad, 2000; Lopez-Otin et al., 2013). Human ageing is associated with characteristic macroscopic changes, which include hair greying, wrinkling of the skin, muscle loss and physical weakness. As individuals age they become more susceptible to a wide range of diseases. In particular, heart disease, cancer, stroke, chronic lower respiratory disease, type 2 diabetes and neurodegeneration are the most common age-associated diseases, and each represents a leading cause of death in aged individuals (Akushevich et al., 2013; Brody and Grant, 2001; Craig et al., 2015; WHO, 2011). Age-associated phenotypes are thought to result from the progressive accumulation of molecular damage, and this phenomenon is postulated to be the consequence of the age-dependent decrease in the force of selection, which fails to remove deleterious mutations that affect aspects of later life (post-fertility) (Bailey et al., 2004; Charlesworth, 2000; Dolle et al., 2000). The age-dependent accumulation of molecular damage induces decreased DNA or protein stability, failure in energy production and utilization, and disruption of homeostasis, leading to structural and functional decay (Lopez-Otin et al., 2013). It is also predicted that mutations providing an overall fitness benefit throughout an organism's lifespan are likely to increase in frequency in a population, even if their phenotypic effect at older ages is detrimental (Williams, 1957).

Human progeroid syndromes and extreme human longevity (see definitions below) offer two biological extremes that have helped to shed light on the basic genetic and physiological mechanisms associated with accelerated ageing and extreme lifespan in humans (Burtner and Kennedy, 2010; Eisenstein, 2012). Human progeroid syndromes are a set of monogenic disorders associated with dysfunctions in the DNA repair machinery or improper formation of the nuclear lamina that lead to premature ageing-like symptoms (Burtner and Kennedy, 2010; De Sandre-Giovannoli et al., 2003; Kitano, 2014; Veith and Mangerich, 2015). Human extreme longevity is a complex phenotype that depends on the interaction between multiple genetic variants and environmental conditions. Importantly, the causal role of different biological mechanisms and genetic variants on human longevity remains elusive owing to obvious experimental limitations with human subjects and the low frequency of centenarians.

Human cell lines provide a great resource for ageing research because they allow the study of several aspects of human cellular biology in a Petri dish (de Magalhaes, 2004; Hashizume et al., 2015); however, they restrict the relevance of the findings to the cellular aspects of individual ageing, and provide limited contribution to the understanding of the *in vivo* mechanisms involved in organismal ageing. An obvious alternative strategy to overcome some of these limitations involves the use of model organisms that either share or mimic the ageing-associated processes of humans.

The use of model organisms has been key to improving the understanding of the molecular mechanisms underlying ageing and the wide spectrum of age-related diseases. The most successful model organisms used in the field of ageing include non-vertebrate models, e.g. yeast, worms and flies, and vertebrate models, e.g. zebrafish and mice. The lifespan of these model organisms in captivity ranges from a few weeks to several years, and the spectrum of their ageing phenotypes is extremely diverse, each mimicking different features associated with human ageing (Table 1). In this article, we first introduce the most commonly adopted model organisms in research on ageing and age-associated diseases. We then detail features of the African turquoise killifish that make it a promising complementary model system for the exploration of ageing *in vivo*, and highlight its potential to provide insight into ageing-related diseases.

**Table 1.**
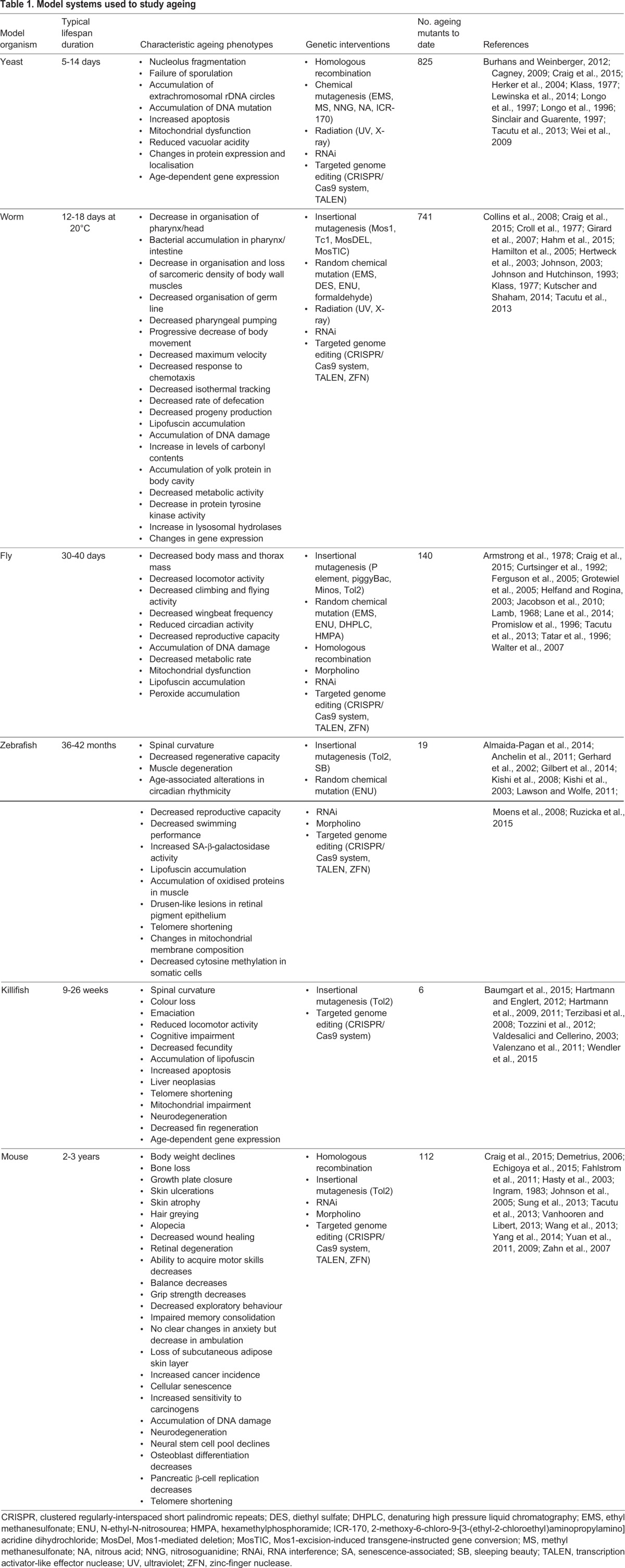
**Model systems used to study ageing**

## Common model organisms used in ageing research

### Non-vertebrate models

The budding yeast (*Saccharomyces cerevisae*) is a unicellular eukaryote that is widely used in ageing research (Henderson and Gottschling, 2008; Herker et al., 2004; Sinclair and Guarente, 1997; Verduyckt et al., 2016). The budding yeast undergoes a limited number of replication events throughout its lifetime. The total number of daughter cells produced by a mother cell defines the yeast cell replicative lifespan (RLS), which is used as a direct measure of yeast survival. Another factor used to measure yeast survival is the survival time of populations of non-dividing yeast cells, which defines the so-called chronological life span (CLS) (Herker et al., 2004; Kaeberlein, 2010; Longo et al., 1996).

Dietary restriction (DR), i.e. reduced food intake without malnutrition (Colman et al., 2014; Piper et al., 2011), is one of the better-studied interventions capable of delaying ageing and prolonging lifespan in several species (Fontana and Partridge, 2015). In line with this, DR has been shown to increase yeast RLS as well as CLS. Some of the most important cellular effectors involved in increasing lifespan belong to the target of rapamycin (TOR) molecular pathway (Bonawitz et al., 2007; Kaeberlein et al., 2005; Powers et al., 2006). This molecular pathway is involved in the regulation of protein synthesis and degradation in response to nutrient quantity and quality (Stanfel et al., 2009), and is largely conserved from yeast to mammals. Importantly, this molecular pathway is impaired in human metabolic dysfunctions such as diabetes and obesity (Cota et al., 2008; Fraenkel et al., 2008).

Given the conservation of the basic eukaryotic intracellular organelles and machinery, yeast has been successfully used as a model to study the intracellular effects of mutated human genes involved in several age-associated neurodegenerative diseases, including Parkinson's and Alzheimer's diseases (Cooper et al., 2006; Khurana and Lindquist, 2010). However, because yeast are unicellular organisms, their use as a model system for ageing is limited to the understanding of the cellular mechanisms. Additionally, some features associated with yeast ageing are specific to yeast biology, such as the age-related accumulation of extrachromosomal ribosomal DNA (rDNA) circles (ERCs) in yeast mother cells (Sinclair and Guarente, 1997). ERCs do not have a direct correlate with the ageing process of other organisms, and demonstrate that, although some ageing-related mechanisms are shared across different taxa, some others are species-specific.

*Caenorhabditis elegans* (*C. elegans*) is a transparent soil nematode that has been utilized as an experimental model system since 1974 (Brenner, 1974). Its usefulness as a model can be in part attributed to the fact that it is a multicellular organism and its life cycle can be entirely recapitulated in a Petri dish. In laboratory conditions, this worm, of about 1 mm in length in the adult form, lives only a few weeks and its life cycle has been thoroughly investigated (http://www.wormatlas.org/). Many ageing phenotypes of the worm are also shared with other organisms, including humans, such as decreased overall body motility and food consumption (measured as pharyngeal pumping rate), progressively increased DNA damage, decreased metabolic activity, accumulation of age-pigments and dramatic changes in age-dependent gene expression (Collins et al., 2008). Interestingly, during development, *C. elegans* can enter a stress-resistant biological state called dauer, characterised by typical morphological changes and the capacity to survive through starvation, temperature changes and other stressors (Gottlieb and Ruvkun, 1994; Lithgow et al., 1995; Riddle et al., 1981). The genes involved in the regulation of dauer formation in *C. elegans* play a key role in regulating worm longevity, and their function in regulating stress responses is conserved across many organisms, including humans (Kenyon et al., 1993). The worm is highly amenable to genetic manipulation (Klass, 1983), which has enabled several genetic screens that have brought to light pathways involved in ageing and lifespan. Large-scale screening of several worm mutant lines and genes (Klass, 1983; Wong et al., 1995; Yang and Wilson, 2000), particularly using RNA interference (RNAi) screening (Hamilton et al., 2005; Lee et al., 2003; Yanos et al., 2012), has been a key contributor to the fundamental insights made into the molecular genetics of ageing. Importantly, these genetic screens provided the first evidence that a single gene can modulate longevity in a multicellular eukaryote (Kimura et al., 1997; Klass, 1983; Lee et al., 2001; Ogg et al., 1997; Tissenbaum and Ruvkun, 1998). These results laid the foundation for the discovery of shared cellular and organismal mechanisms, such as the stress response and nutrient-sensing, that control longevity in multiple organisms, and these findings are likely to be relevant to human ageing (Kenyon, 2010; Kim, 2007; Lopez-Otin et al., 2013).

The fruit fly, *Drosophila melanogaster* (*D. melanogaster*), is a well-established model organism, with many key attributes that make it a valuable experimental system, such as being easy to maintain and amenable to manipulation using advanced genetic tools. In addition, the fruit fly community benefits from the availability of public resources, including mutant and gene libraries (http://flybase.org/) (Matthews et al., 2005; Millburn et al., 2016). Wild-type *D. melanogaster* can survive for just a few months in captivity, and it is therefore an excellent experimental model in which to study the biology of ageing and the effects of different interventions on overall life expectancy. Although modern flies and worms are phylogenetically equally related to vertebrates (Masoro and Austad, 2006), flies have some features that make them more closely resemble higher vertebrates, such as a complex and centralised brain, a heart (Chintapalli et al., 2007), and the presence of multipotent adult stem cells in the mid-gut and the gonads (Micchelli and Perrimon, 2006; Ohlstein and Spradling, 2006; Wallenfang et al., 2006). Their functional proximity to vertebrates has allowed the development of several fly models that are useful for the study of mammalian ageing, in particular the effects of ageing on muscle, brain, cardiac and intestinal tissues (Demontis and Perrimon, 2010; Guo et al., 2014; Haddadi et al., 2014; Ocorr et al., 2007).

The availability of rapid and effective molecular, biochemical and genetic tools for application in invertebrate models has provided a great advantage in the field of ageing. Indeed, research using these models has yielded important insights into the basic cellular and molecular mechanisms that underlie the ageing process in a wide range of living organisms. However, invertebrate model organisms do not allow exploration of all aspects of human ageing. For instance, the currently available invertebrate model organisms (i.e. worms and flies) are not ideal platforms to study the process of tumorigenesis and cancer during ageing, because adults of these organisms are mostly composed of post-mitotic cells, i.e. cells that no longer undergo the cell cycle and therefore cannot develop cancer (Masoro, 1996). Additionally, although they are equipped with an effective innate immune system, invertebrates lack a vertebrate-like lymphocyte-based adaptive immune system (Johnson, 2003). In addition, the lack of an endoskeleton and a spinal cord precludes their use as models to study ageing in the bone systems or in the spinal cord (Ethan and McGinnis, 2004; Tissenbaum, 2015). For these reasons, vertebrate model organisms, which are more physiologically similar to humans, are invaluable model organisms to study a wider range of ageing-specific phenotypes that affect humans.

### Vertebrate models

Zebrafish (*Danio rerio*) has become a tremendously successful vertebrate experimental model organism, and it is currently widely used by a large scientific community. Zebrafish have transparent embryos, making them particularly amenable to live imaging studies in the early stages of development. Zebrafish have lower maintenance costs compared to mice, produce many embryos, and are amenable to large-scale genetic and pharmacological interventions. Based on these advantages, research on zebrafish has provided a fundamental contribution to the basic understanding of the molecular mechanisms underlying vertebrate development (Duboc et al., 2015; Kikuchi, 2015; Veldman and Lin, 2008). In addition, owing to their capacity of regenerating the heart, tail and spinal cord (Asnani and Peterson, 2014; Becker et al., 1997; Poss et al., 2003, 2002), they are also used as models for regenerative medicine (Goessling and North, 2014). These attributes have naturally paved the way for zebrafish to also be introduced as a model for studying ageing (Anchelin et al., 2011; Gilbert et al., 2014; Kishi, 2011; Kishi et al., 2003; Van Houcke et al., 2015). The model displays key hallmarks of ageing, including age-dependent mitochondrial dysfunction, telomere deterioration (loss of the terminal portions of the chromosomes) and protein oxidation (Kishi et al., 2003). Telomerase-deficient transgenic zebrafish (Anchelin et al., 2013; Henriques et al., 2013) (with a median lifespan of 9 months) show accelerated ageing phenotypes, demonstrating the important role of telomere length and stability in regulating vertebrate ageing and lifespan.

The laboratory mouse, *Mus musculus*, is the most widely adopted model system to investigate the biology of mammalian ageing. Given that the basic physiological mechanisms are highly conserved between mice and humans, the laboratory mouse has helped reveal many of the causal molecular mechanisms connecting ageing and ageing-related diseases (Liao and Kennedy, 2014; Vanhooren and Libert, 2013). The mouse ageing phenome includes up to 32 available inbred strains (Yuan et al., 2011), and the lifespan of the available inbred mouse strains varies from 2 to 4 years. The most commonly used strain in ageing research, C57BL/6, has a median lifespan of 914 days (Yuan et al., 2011). Such a short lifespan for a mammal makes it possible to test, in a relatively short time, the effects of any genetic, pharmacological or environmental intervention on mammalian ageing and lifespan. However, maintenance costs for mice are much higher compared to other model animals used to study ageing (Lieschke and Currie, 2007), and its lower litter size can limit opportunities for high-throughput screening under variable genetic, environmental and pharmacological interventions.

Importantly, the ageing vertebrate experimental model organisms listed above are characterised by a much longer lifespan than the invertebrate model organisms, substantially impacting experimental duration and costs. Therefore, there is a need to develop new model systems that share the advantages of short-lived non-vertebrate model organisms but demonstrate the physiological proximity to humans of vertebrate model organisms.

The African turquoise killifish (*Nothobranchius furzeri*), a teleost fish with a natural lifespan ranging between 4 and 9 months, is emerging as a new promising model organism in ageing research (Genade et al., 2005; Terzibasi et al., 2008). Below, we summarise the major advantages that make this species a competitive new model organism for ageing research. We describe its development, life cycle, known ageing phenotypes and the available approaches for modulating its lifespan experimentally, as well as the recent advances in transgenesis techniques and their applications in this animal. Finally, we discuss possible future applications of the turquoise killifish to understanding human ageing and ageing-associated diseases.

## Turquoise killifish in nature and in the laboratory

In nature, the turquoise killifish dwells in seasonal water bodies in Mozambique and Zimbabwe (Reichard et al., 2009). Its habitat is yearly characterised by a brief rainy season followed by a longer dry season. During the rainy season, ephemeral ponds form along seasonal river drainages. The fish then rapidly hatch, reach sexual maturity in less than a month and reproduce before the water completely dries out in the subsequent dry season. The embryos are uniquely adapted to survive and develop in dry mud during the dry season (Blazek et al., 2013; Furness et al., 2015).

**Fig. 1.**
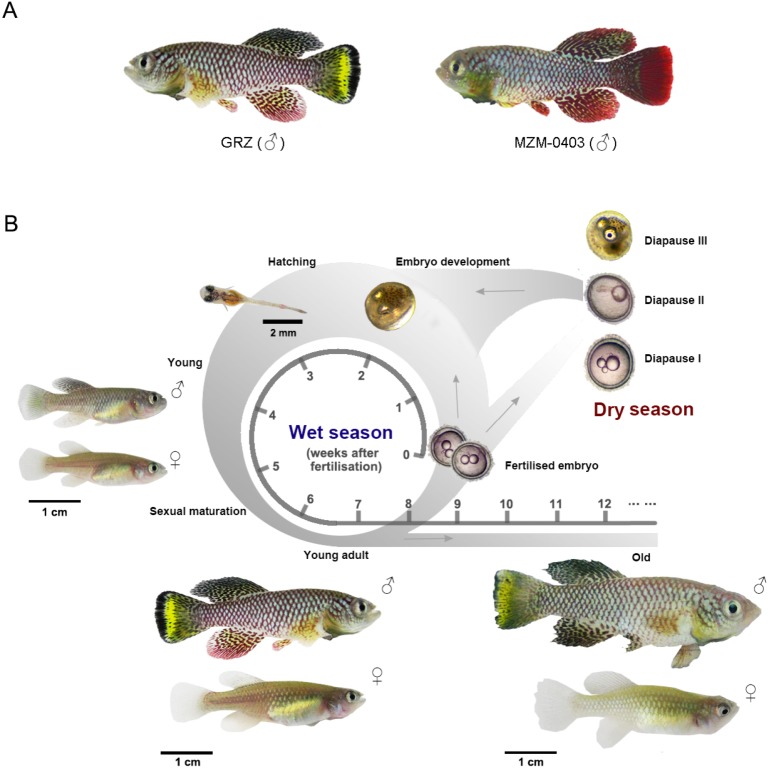
**Captive strains and the** **life cycle of the turquoise killifish.** (A) Commonly used laboratory strains of the turquoise killifish: the short-lived strain (left; GRZ, yellow-tailed male fish) and long-lived strain (right; MZM-0403, red-tailed male fish). (B) Turquoise killifish life cycle (the time scale is based on the short-lived laboratory strain). Embryos can enter normal development or a developmentally arrested state called diapause, which lasts from a few weeks to several months and protects killifish during the dry season in the wild. Diapause consists of three different stages called diapause I, II and III. During the wet season in the wild (see main text) – and in laboratory conditions – hatched fry fully develop within 3-4 weeks and start spawning. Male fish are larger than females and have colourful fins and body, whereas the female fish are dull. Upon ageing (‘old’), fish lose body colour, fin structure deteriorates and the spine becomes bent. The age for young, young adult and old fish is indicated in weeks.

The current turquoise killifish laboratory strains include the inbred ‘GRZ’ strain, derived from an original population collected in 1968 (Genade et al., 2005; Valdesalici and Cellerino, 2003), and several wild strains that were derived more recently (Bartakova et al., 2013; Reichwald et al., 2009; Terzibasi et al., 2008). The GRZ strain has the shortest recorded lifespan among all the available turquoise killifish strains, with a median lifespan ranging from 9 to 16 weeks depending on the culture conditions (Genade et al., 2005; Kirschner et al., 2012; Terzibasi et al., 2008) (Fig. 1A, left), whereas longer-lived strains have median lifespans ranging from 23 to 28 weeks (Kirschner et al., 2012; Terzibasi et al., 2008; Valenzano et al., 2015) (Fig. 1A, right). Interestingly, although, in captivity, no difference in killifish survival is observed between the sexes (Valenzano et al., 2015, 2006b), large differences in sex ratios are observed in the wild, where females tend to be more frequent than males, which is also observed to some extent in other species, including humans (Reichard et al., 2009).

### Life cycle of the turquoise killifish

The life cycle of the turquoise killifish is relatively unique because they are adapted to reach sexual maturity and reproduce during a very short (wet) period (Cellerino et al., 2015). Once hatched, fish grow very rapidly, as they need to complete sexual maturation and reproduce before the water completely evaporates (Podrabsky, 1999) (Fig. 1B). Each captive turquoise killifish female lays 20-40 eggs per day, with a maximum recorded number of 200 (Blazek et al., 2013; Graf et al., 2010; Polacik and Reichard, 2011), which is a similar order of magnitude as zebrafish (Eaton and Farley, 1974a,b; Hisaoka and Firlit, 1962; Kalueff et al., 2014), although zebrafish reproduce for a longer period. Although zebrafish and killifish can be bred in similar ways, and their embryo size is comparable, embryonic development in the turquoise killifish is slower (∼2-3 weeks) than in zebrafish (∼2-3 days) (Dolfi et al., 2014; Kimmel et al., 1995). However, owing to their rapid sexual maturation, which in killifish takes 3-4 weeks in captivity (Fig. 1B), the complete life cycle under controlled laboratory conditions is faster in the turquoise killifish (5.5-8 weeks) than in zebrafish (12-13 weeks) (https://zfin.org/) (Avdesh et al., 2012). The embryo development in the turquoise killifish is characterised by a developmentally arrested state called diapause (Wourms, 1972), in which embryos can survive over several months in the absence of water, encased in dry mud (Reichard et al., 2009). This special developmental state is functionally analogous to the *C. elegans* dauer state. Under controlled laboratory conditions, killifish embryos can skip diapause and therefore develop rapidly (Furness et al., 2015; Valenzano et al., 2011). Maternal age and temperature of embryo incubation are important factors regulating the exit from diapause in the killifish (Markofsky and Matias, 1977; Podrabsky et al., 2010). However, the molecular mechanisms involved in entry, persistence and exit from diapause are still largely uncharacterised in killifish. Because the genes controlling larval dauer in *C. elegans* have been shown to be crucial in regulating adult phenotypes, including ageing, it is important to identify what genes regulate killifish diapause and test whether they can also play a role in regulating adult physiology and ageing phenotypes, including ageing-related diseases. If the genes regulating killifish diapause also regulate adult phenotypes, including ageing, they could be relevant for understanding ageing-related phenotypes in humans.

### Ageing phenotypes in the turquoise killifish

Despite its relatively short lifespan for a vertebrate, the turquoise killifish shows many molecular, cellular and physiological ageing phenotypes that are shared with many other organisms, including humans (Genade et al., 2005; Hartmann et al., 2009, 2011; Terzibasi et al., 2009, 2007; Valenzano et al., 2006b). Similarly to ageing mammals, who progressively lose hair and skin pigment with age (Geyfman and Andersen, 2010), male turquoise killifish – which are more colourful than females – progressively lose body and tail colour as well as their distinct patterning as they age (Fig. 1B). Old age in this short-lived vertebrate is also associated with abnormal spine curvature, defective vision, fin structure deterioration, decreased spontaneous locomotion activity, learning impairment (Genade et al., 2005; Valenzano et al., 2006b) and, interestingly, an increased risk of cancer (Baumgart et al., 2015). Fecundity also declines with age in the turquoise killifish, with embryo production reaching a peak at around 8-10 weeks and gradually declining thereafter (Blazek et al., 2013). These macroscopic phenotypes recapitulate several of the complex age-dependent changes that occur in other vertebrates, including mouse and humans (Vanhooren and Libert, 2013). Compared to other fish, the striking feature of killifish ageing is its rapid onset within 3-4 months of age. For comparison, in zebrafish studies, individuals over 24 months of age are considered by researchers working on zebrafish models of ageing to be ‘old’ (Kishi et al., 2003).

Several ageing biomarkers have been developed to characterise the physiological age of killifish. Lipofuscin, a yellow-brown autofluorescent pigment whose concentration increases with age in several species, including humans (Goyal, 1982), accumulates in the brain and liver of old killifish (Goyal, 1982; Terzibasi et al., 2008, 2007). Senescence-associated β-galactosidase (SA-β-gal) staining, a marker for cellular senescence and stress response in human cells (Cristofalo, 2005; Dimri et al., 1995; Kurz et al., 2000; Untergasser et al., 2003; Yegorov et al., 1998), significantly increases in the skin of aged fish (Terzibasi et al., 2007). Neurodegeneration – measured by Fluoro-Jade B, which stains cell bodies, dendrites and axons of degenerating neurons but not those of healthy neurons (Schmued and Hopkins, 2000) – increases in fish brains from as early as 2 months of age, strongly suggesting a spontaneous age-dependent increase in neurodegeneration (Terzibasi et al., 2007; Valenzano et al., 2006b). The availability of various ageing biomarkers for the turquoise killifish allows characterisation of age-related changes in many tissues and under different experimental conditions, further facilitating the use of this model system for ageing research.

Neoplastic lesions have been measured in turquoise killifish strains using several tumour-associated proteins, including Bcl-2, cytokeratin-8, carcinoembryonic antigen and mutated p53 (Di Cicco et al., 2011). In this study, the liver showed the highest rate of tumour incidence, and the short-lived GRZ strain showed an earlier onset of liver tumours compared to longer-lived strains. Additionally, the occurrence of these lesions increases as the fish aged (Di Cicco et al., 2011; Pompei et al., 2001), which is also observed in humans (de Magalhaes, 2013). Finally, liver cancer in killifish has a higher incidence in males than in females (Di Cicco et al., 2011), and this also replicates the pattern of liver cancer incidence in humans (Nordenstedt et al., 2010).

A range of factors could underlie liver cancer development in killifish, including chronic liver infection, metabolic perturbations (e.g. in carbohydrate metabolism) or genetic predisposition. It is of pivotal importance to compare cancer incidence in captive-bred and wild killifish populations to evaluate the relative contributions of environmental and genetic factors to this phenotype. Although liver cancer is not among the most common cancers in humans, it is one of the leading causes of cancer-related deaths worldwide (globocan.iarc.fr/Pages/fact_sheets_population.aspx). There are several mouse models of hepatocellular carcinoma, in which the condition can be induced chemically or genetically (Bakiri and Wagner, 2013). The distribution of the lesions and neoplasias in aged killifish, as gleaned from post-mortem analyses, largely differs from that in humans, mice and zebrafish (Di Cicco et al., 2011), indicating that mortality in killifish might be driven by cancer more than it is in other, longer-lived organisms. This, together with the fact that there are relatively few good models for these types of cancer in humans, highlight the potential to develop the turquoise killifish as a complementary model for liver cancer.

Age-dependent telomere attrition has been linked to organismal ageing in humans and in many other model organisms (Benetos et al., 2001; Harley et al., 1990; Lopez-Otin et al., 2013). Interestingly, killifish telomeres, which are over four times shorter than in mice (Zhu et al., 1998), are comparable in length to human telomeres (Hartmann et al., 2009). Although telomeres have been shown to shorten with age in a long-lived turquoise killifish strain, they did not shorten significantly in the short-lived GRZ strain, possibly owing to the very short lifespan of this strain (Hartmann et al., 2009). This suggests that telomere attrition might not contribute to rapid ageing of the short-lived GRZ strain. However, a telomerase mutant line of the turquoise killifish showed premature infertility, a dramatic decrease of red and white blood cell numbers, abnormalities in the epithelial cells of the intestine, including decreased polarity, and increased nuclear/cytoplasmic ratio (Harel et al., 2015). These results suggest that telomerase plays a key role in the maintenance of organismal homeostasis in the turquoise killifish, as in other organisms.

Mitochondrial DNA (mtDNA) instability has been associated with ageing in many species, including humans (Barazzoni et al., 2000; Lopez-Otin et al., 2013; Tauchi and Sato, 1968; Yen et al., 1989; Yui et al., 2003). mtDNA lacks protective histones; therefore, it is more likely to accumulate mutations compared to nuclear DNA (Tatarenkov and Avise, 2007). mtDNA mutations and deletions increase with age, whereas mtDNA copy number decreases, especially in the liver of mouse, rat and humans (Barazzoni et al., 2000; Bratic and Larsson, 2013). The increased mtDNA mutations and deletions with age drive an early onset of age-associated phenotypes (Vermulst et al., 2008). In line with this, mtDNA copy number was found to be significantly reduced in several turquoise killifish tissues, including brain, liver and muscle, and its biogenesis was also impaired in muscles from old individuals (Hartmann et al., 2011).

The capacity to regenerate and repair tissues and organs helps to ensure homeostasis, resulting in the maintenance of an optimal health status. Interestingly, a recent study has shown that the capacity to regenerate the caudal fin after amputation was progressively impaired throughout ageing in the long-lived MZM-0703 turquoise killifish strain (Wendler et al., 2015). Whereas 8-week-old fish are capable of regenerating the caudal fin almost completely (98%) within 4 weeks, fish at 54 weeks of age could regenerate only 46% of the original fin size (Wendler et al., 2015).

## Experimental modulation of the lifespan of turquoise killifish

Ageing is a highly integrated process that can be experimentally modulated by various types of interventions, including changes in nutrients, drugs, temperature and social conditions, as well as genetic modifications.

As mentioned above, DR can effectively modulate lifespan and ageing in several organisms, ranging broadly from yeast to worms, flies, beetles, chicken, mice, rats, dogs and macaques (Fontana and Partridge, 2015; Taormina and Mirisola, 2014). The effects of DR on lifespan and ageing biomarkers were tested in the turquoise killifish, which were fed every other day instead of daily (Terzibasi et al., 2009). The results were strain-dependent: DR resulted in prolonged lifespan in the short-lived GRZ strain but not in a wild-derived, long-lived MZM-0410 strain (Terzibasi et al., 2008). Under the DR regimen, the short-lived strain showed reduced neurodegeneration, slower accumulation of lipofuscin, improved learning performance and decreased occurrence of tumours (Terzibasi et al., 2009). This indicates that DR via every-other-day feeding can delay ageing in the turquoise killifish, depending on the genetic background. The effects of DR need testing under different nutrient restriction paradigms, including overall reduction of daily nutrient intake, or by varying the contribution of different micronutrients in the diet, which has shown to be effective in modulating ageing and longevity in other model organisms (Mair et al., 2005; Miller et al., 2005; Skorupa et al., 2008; Solon-Biet et al., 2015; Zimmerman et al., 2003). The effects of different nutrient restriction paradigms on humans are still largely unknown, warranting further investigation in the context of model systems, including the turquoise killifish.

Temperature is an important factor that has a huge effect in modulating metabolic rate and organism physiology (Conti, 2008; Lithgow, 1996). Modulating both environmental and individual temperature has a significant impact on organism physiology and can, within a specific range, modulate lifespan and ageing in many model organisms (Conti et al., 2006; Lamb, 1968; Liu and Walford, 1966; Mair et al., 2003). Decreased water temperature is sufficient to extend both the median (1 week) and maximum (1.5 weeks) lifespan of turquoise killifish, and leads to a 40% decrease in adult size compared to adult fish grown under regular culturing temperature, indicating a dramatic influence of temperature on metabolism. Several age-associated phenotypes, such as lipofuscin accumulation, spontaneous locomotor activity and learning performance, are also significantly improved in fish cultured at a lower temperature (Valenzano et al., 2006a). Importantly, previous studies performed in fish (Atlantic salmon and *Cynolebias adloffi*) showed that temperature changes can modulate lifespan and growth in different directions, and that there exist temperature optima for lifespan extension and growth optimisation (Handelanda et al., 2008; Liu and Walford, 1966). However, studies that accurately correlate a broad range of temperatures with fish growth rates, survival and reproduction are still lacking, and these are needed to establish a clear mechanistic connection between environmental temperature, metabolism and life history. The understanding of the molecular mechanisms responsible for lifespan extension through temperature modulation could be used in the future to design molecular interventions that slow down ageing, retard the onset of age-related pathologies and ultimately extend lifespan.

The use of the natural polyphenol resveratrol, known to increase lifespan and delay ageing in worms and flies, can increase median and maximum lifespan in a dose-dependent manner in both male and female turquoise killifish. Compared to control-fed fish, resveratrol-fed fish remained physically active for a longer time, indicating that this compound is sufficient to retard the age-dependent decline in physical activity. Similarly, resveratrol-fed fish showed better learning performance at later ages than control-fed fish (Genade and Lang, 2013; Liu et al., 2015; Valenzano and Cellerino, 2006; Valenzano et al., 2006b; Yu and Li, 2012). These effects are consistent with the biological effect of resveratrol on ageing and age-associated physiology in yeast, worms, flies and mice fed a high-fat diet (Baur et al., 2006; Marchal et al., 2013). Studies linking the effects of resveratrol on human metabolism and ageing are to date inconclusive and more work is needed to clarify the effects of this natural compound in our species.

The modulation of the turquoise killifish lifespan and ageing through external interventions such as diet, temperature and chemicals supports the use of this organism as an experimental platform for large-scale screens of age-modulating genes and chemicals.

## Genetic modifications in the turquoise killifish

**Fig. 2.**
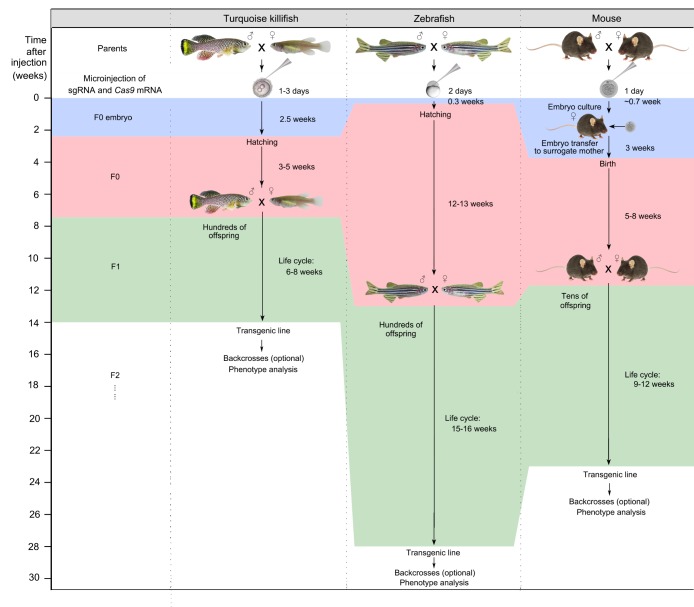
**Side-by-side comparison of timing of transgenic line generation using genetic manipulations in the turquoise killifish, zebrafish and mouse.** Synthesised single guide RNA (sgRNA) and Cas9 nucleases are injected into one-cell-stage embryos. Injected embryos are called F0 embryos. After hatching, transgenic fish are backcrossed to wild-type fish and generate F1 offspring. Further backcrosses are used to remove off-target mutations. Data are from the following references: turquoise killifish (Harel et al., 2015); zebrafish (Hwang et al., 2013; Jao et al., 2013); mouse (Wang et al., 2013).

To date, two methods have been successfully developed to modify the turquoise killifish genome: random genome integration through the Tol2 DNA transposase and targeted genome editing using CRISPR/Cas9 nuclease. To elicit genome editing, both methods require microinjection of RNA and DNA combinations into the one-cell-stage embryo (Table 1 and Fig. 2) (Harel et al., 2015; Hartmann and Englert, 2012; Valenzano et al., 2011). Whereas the Tol2 system was initially applied as a proof-of-principle to validate the possibility of generating transgenic turquoise killifish, CRISPR/Cas9 has recently been established in this species to directly test the role of genes involved in key ageing regulatory pathways (Harel et al., 2015). Deletion mutants or variants in relevant ageing pathways, including telomere attrition, deregulation of nutrient sensing, loss of proteostasis, genomic instability, mitochondrial dysfunction, epigenetic alteration, altered intercellular communication, cellular senescence and stem cell exhaustion, have been generated (Harel et al., 2015). At each cell division, telomeres cannot be fully replicated, and consequently shorten. This phenomenon of telomere deterioration progresses with ageing. Importantly, the loss of telomere length maintenance is crucial for cancer and degenerative diseases (Aubert and Lansdorp, 2008; Lopez-Otin et al., 2013). Telomerase reverse transcriptase (TERT) is a crucial protein whose key role is to elongate telomeres by adding nucleic acids (Aubert and Lansdorp, 2008). In humans, telomere dysfunction induces many degenerative diseases, including dyskeratosis congenita and pulmonary fibrosis. Harel et al. generated several *TERT* mutants in the turquoise killifish using the CRISPR/Cas9 nuclease system, and the deletion mutants lacking the catalytic function of TERT underwent age-dependent telomere shortening (Harel et al., 2015). In addition, *TERT* mutant fish developed atrophied gonads and severe age-related morphological defects in actively proliferating tissues such as testes, intestine and blood, compared to less actively proliferating tissues such as heart, muscle, liver and kidney (Harel et al., 2015). The non-synonymous variant of *TERT* corresponding to human dyskeratosis congenita was successfully generated by homology-directed repair accompanied by the CRISPR/Cas9 nuclease system, showing that this strategy can be effectively used to generate fish models of human-specific diseases (Harel et al., 2015).

As well as engineering the genome of turquoise killifish, direct injection of synthetic RNA into the one-cell-stage embryo recently enabled the development of a turquoise killifish fluorescence ubiquitination cell cycle indicator (FUCCI) (Dolfi et al., 2014). This method takes advantage of two fluorescent-tagged proteins that differentially degrade during the cell cycle, resulting in the accumulation of red fluorescence at the G1 phase of the cell cycle and of green fluorescence at the S/G2/M phases (Sakaue-Sawano et al., 2008). FUCCI expression remains stable for 3-4 days after the FUCCI mRNA injection into killifish embryos and allows the temporal tracking of early cell-division kinetics. When stable FUCCI transgenic killifish lines become available, this tool will become important in monitoring cellular proliferation during development, tumour formation, tissue/organ regeneration and senescence.

Genetic modifications in the turquoise killifish are rapid and highly efficient, permitting generation of stable transgenic lines more rapidly than in any other available vertebrate model (Fig. 2). Combined with their other advantageous features, as described above, efficient genetic manipulation of the turquoise killifish could allow it to become a powerful and highly scalable platform to study age-related changes and diseases (Table 2).

**Table 2.**
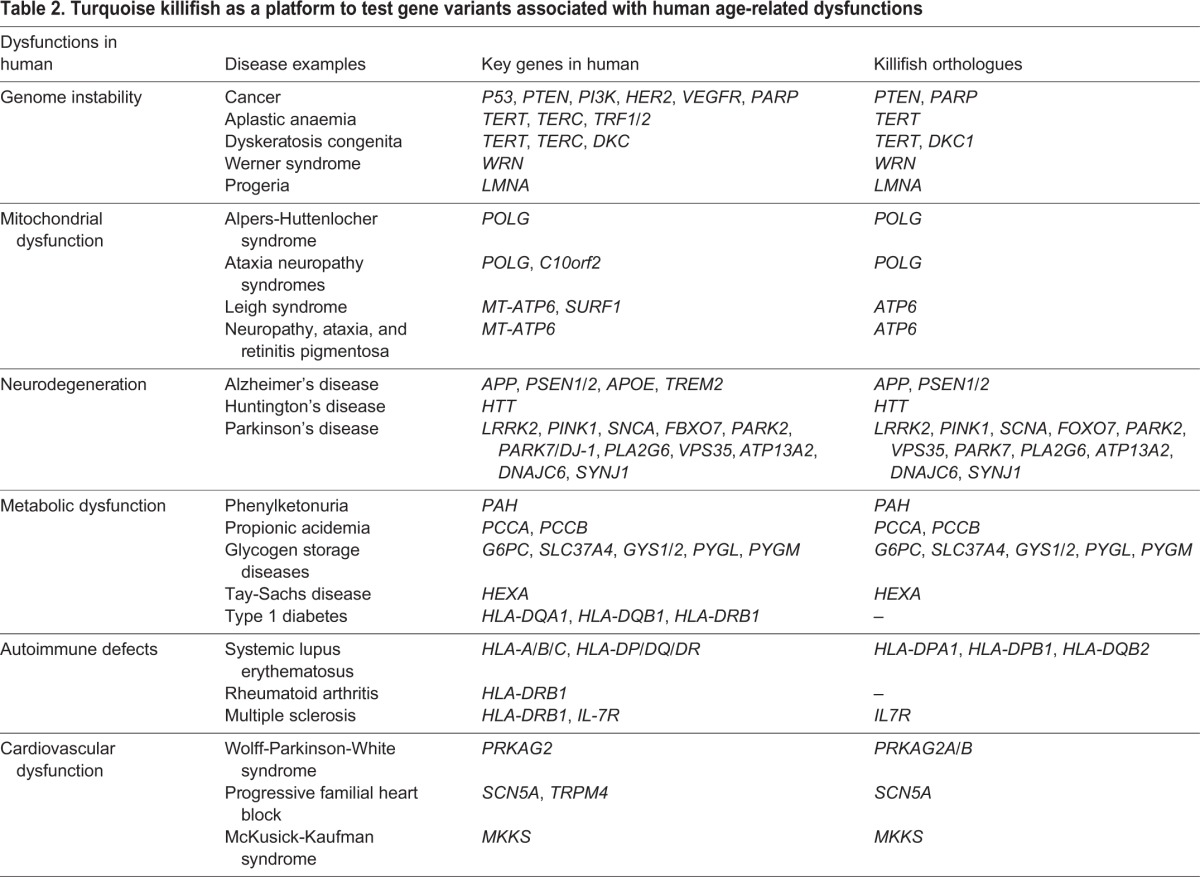
**Turquoise killifish as a platform to test gene variants associated with human age-related dysfunctions**

## Looking forward: potential applications and limitations of killifish models of ageing

The long lifespan of the currently available vertebrate model organisms is a major limitation for the feasibility of large-scale ageing studies. The development of new model organisms is vital to compensate for the limitations of previous model systems. In the past, several fish species have been adopted as potential ageing model organisms, including medaka (Ding et al., 2010; Egami and Eto, 1973; Gopalakrishnan et al., 2013), guppies (Reznick et al., 2006; Woodhead and Pond, 1984; Woodhead et al., 1983) and both American as well as African killifish (Liu and Walford, 1966; Markofsky and Milstoc, 1979a,b). As highlighted in this article, the turquoise killifish has emerged as a promising new model organism for vertebrate ageing because it uniquely combines a short lifespan and life cycle with vertebrate-specific features that are missing from the currently used non-vertebrate model organisms. In particular, it undergoes continuous adult cellular proliferation, has adult stem cells in many tissues and an adaptive immune system, and shows a spontaneous age-dependent increased risk for cancer. The turquoise killifish has the shortest lifespan among all vertebrate models in captivity, and shares several age-associated phenotypes with other vertebrates, including humans (Table 1). Recent genomic and transcriptome data analysis in the turquoise killifish have revealed many orthologous genes to humans and other model organisms (Harel et al., 2015; Petzold et al., 2013; Reichwald et al., 2009). Additionally, the fully sequenced, assembled and annotated genome is now available, together with a high-density Rad-Seq genome map, as well as ChipSeq and transcriptome data from several tissues (Reichwald et al., 2015; Valenzano et al., 2015). The availability of these genomic tools will enable a broad scientific community to take advantage of this model system in an unprecedented way. The turquoise killifish captive strains are highly fecund, facilitating transgenic line generation and genetic screenings (Fig. 2). Importantly, there exists a highly inbred turquoise killifish strain (GRZ) that enables easy testing of the effects of biochemical or genetic modifications on organismal physiology. Thus, the turquoise killifish is likely to provide a very useful platform for testing the function of genes involved in longevity regulation, particularly to examine the underlying genes of extreme longevity. Unique gene variants have been found in human centenarians, as well as very long-lived model organisms such as the naked mole rat and bowhead whale (Keane et al., 2015; Kim et al., 2011; Sebastiani et al., 2011; Willcox et al., 2006). However, their causal role in ageing and longevity is largely unknown. Given the long lifespan of mice and zebrafish, it is impractical to functionally analyse such gene variants, especially when the expected outcome is lifespan extension. The turquoise killifish could fill the need for a short-lived vertebrate, enabling variants linked to extreme longevity to be tested in a rapid and effective way. In parallel, the turquoise killifish allows rapid testing – in vertebrates – of the genetic variants identified in worms and flies (de Magalhaes and Costa, 2009; Tacutu et al., 2013), enabling the translation of findings from short-lived invertebrates to vertebrates.

Human ageing is associated with an increased risk of several diseases, such as cancer or Alzheimer's disease (Table 2), which have a strong genetic component. The turquoise killifish provides the opportunity to test, in a short time, the causal role of specific gene variants in the onset of age-associated diseases. For instance, to assess the effects of a given genetic manipulation on lifespan would be six times faster in killifish than in mice (Fig. 2).

Outbred turquoise killifish strains are used to identify the genetic architecture of naturally occurring phenotypes that differ among strains, such as male colouration, susceptibility to pigment aberration, and survival. Quantitative trait locus (QTL) mapping studies in the turquoise killifish have revealed genomic loci that are significantly associated with such phenotypes (Kirschner et al., 2012; Valenzano et al., 2009). Previous studies in sticklebacks have used genetic mapping to identify the genetic basis of human phenotypic variation (Miller et al., 2007). The possibility to combine genetic mapping with transgenesis in the turquoise killifish could help reveal the basic molecular mechanisms underlying the susceptibility to early ageing and age-related diseases, which might be shared with humans.

Reverse genetic tools, which use transposases and nuclease-based systems to introduce genomic integrations and deletions, were recently developed in the turquoise killifish (Allard et al., 2013; Harel et al., 2015; Hartmann and Englert, 2012; Valenzano et al., 2011). However, forward genetic approaches have not been explicitly tested in the turquoise killifish yet. However, the use of the Tol2 transposase, which introduces portions of DNA in random regions of the host genome – similar to insertional mutagenesis – demonstrates the feasibility of forward genetic applications in this species (Valenzano et al., 2011). Random mutagenesis, using various mutagens such as N-ethyl-N-nitrosourea (ENU), has been used for a long time in many species – from bacteria to yeast, plants and animals – as a powerful method to stably alter the genetic information of an organism, and then to subsequently identify a causal connection between genetic modification and a specific biological phenotype (Muller, 1927), including longevity (Caspary, 2010; Hardy et al., 2010; Jorgensen and Mango, 2002). Several strategies for large-scale screening after chemical-induced mutagenesis in zebrafish have been developed (for a review, see Patton and Zon, 2001), which have resulted in a vast collection of zebrafish mutant lines, each having a specifically altered phenotype (Driever et al., 1996; Haffter et al., 1996; Lawson and Wolfe, 2011; Wang et al., 2012; Westerfield, 2000). Owing to the relevance of the turquoise killifish to study adult phenotypes, mutagenised fish should be screened for the phenotype of interest after sexual maturation. Unlike embryo or larval screens, which can take advantage of high population densities of individuals and rapid experimental time, adult screens – in particular those aimed at selecting longer-lived individuals – require a larger space and take longer. Even though turquoise killifish males can be territorial – especially when grown in isolation – this species allows for survival screens in group-housed conditions. Experiments to establish the effect of fish population density on experimental survival are currently ongoing in the Valenzano lab. However, the application of random mutagenesis to screen for turquoise killifish mutants that live longer requires large breeding facilities. In fact, screening for longevity mutants requires that all of the mutagenised fish have to breed, since by the time they breed it is not possible to assess whether they will be long-lived or short-lived. Only the offspring of the long-lived mutants will be used to generate mutant fish lines. An alternative strategy, widely employed in the *C. elegans* and *D. melanogaster* fields to identify genes involved in the regulation of longevity, is to induce mutations and then screen juvenile fish for stress resistance against chemicals or physical stressors (Denzel et al., 2014; Lin et al., 1998; Walker et al., 1998). This alternative strategy might help to overcome spatial and temporal limitations of longevity screening using exclusively adult turquoise killifish. The application of random mutagenesis accompanied by accessible phenotypic analyses of the turquoise killifish has not yet been developed but has great potential to reveal currently unknown molecular mechanisms of ageing and age-associated diseases. The turquoise killifish also has the potential to be used for high-throughput drug screening in a similar way to zebrafish (Gibert et al., 2013), with the advantage of a shorter lifespan and life cycle. This application would make it an ideal system to test the effects of drugs on adult-specific phenotypes involved in ageing, age-related diseases and overall longevity.

Although model organisms in ageing research have greatly improved our understanding of the shared features of the ageing process across organisms, no single model is a perfect model of human ageing. It is important to note the intrinsic limitations of the turquoise killifish. For example, it cannot be directly used to study organs that are not shared between fish and mammals, such as lungs or bone marrow. Additionally, being a novel model organism, it still lacks important community-based research resources, such as a large-scale stock centre with wild-derived, transgenic and mutant strains, which are readily available in other model organisms that are more established. However, molecular tools are rapidly developing in the turquoise killifish, including several linkage maps (Kirschner et al., 2012; Valenzano et al., 2015, 2009), a transcriptome atlas (Petzold et al., 2013), and a completely sequenced, assembled and annotated genome (Reichwald et al., 2015; Valenzano et al., 2015). The short lifespan of this species, one of its biggest strengths as a vertebrate model organism, could potentially also constitute a limitation for its applications as a ‘natural ageing’ model system, because some of the very late-age phenotypes occurring for instance in humans (e.g. cardiovascular disease and dementia) might be not shared in this species. There are, however, several strains within this species, some of which differ substantially in captive lifespan, and a comparative study of age-dependent phenotypes in multiple strains could be used as an approach to investigate a larger range of such phenotypes. Additionally, other species of the same genus, which are longer-lived than the turquoise killifish, are available and can be housed under conditions similar to the turquoise killifish (Genade et al., 2005; Terzibasi et al., 2008), offering an additional opportunity for the comparative study of the molecular and genetic basis of vertebrate ageing.

In summary, the short-lived turquoise killifish has the potential to become a central model system in the field of the biology of ageing and be used as a bridge from the short-lived invertebrates to the longer-lived mammalian models in the study of the biological mechanisms that underlie age-associated diseases (Table 2). The growing scientific community working on the turquoise killifish is constantly developing novel genetic tools and a wide set of resources, which we predict will build this model up as a strong platform for the discovery of novel basic mechanisms that play an important role in vertebrate ageing.
